# Static Calibration of a New Three-Axis Fiber Bragg Grating-Based Optical Accelerometer

**DOI:** 10.3390/s25030835

**Published:** 2025-01-30

**Authors:** Abraham Perez-Alonzo, Luis Alvarez-Icaza, Gabriel E. Sandoval-Romero

**Affiliations:** 1 Instituto de Ingeniería, Universidad Nacional Autónoma de México, Mexico City 04510, Mexico; alvar@pumas.iingen.unam.mx; 2 Instituto de Ciencias Aplicadas y Tecnología, Universidad Nacional Autónoma de México, Mexico City 04510, Mexico; eduardo.sandoval@icat.unam.mx

**Keywords:** sensor characterization, static calibration, FBG-based accelerometer, overlapping interrogation method

## Abstract

Optical sensors are a promising technology in structural and health monitoring due to their high sensitivity and immunity to electromagnetic interference. Because of their high sensitivity, they can register the responses of buildings to a wide range of motions, including those induced by ambient noise, or detect small structural changes caused by aging or environmental factors. In previous work, an FBG-based accelerometer was introduced that is suitable for use as an autonomous unit since it does not make use of any interrogator equipment. In this paper, we present the results of the characterization of this device, which yielded the best precision and accuracy. The results show the following: (i) improvements in the orthogonality of the sensor axes, which impact their cross-axis sensitivity; (ii) reductions in the electronic noise, which increase the signal-to-noise ratio. The results of our static characterization show that, in the worst case, we can obtain a correlation coefficient R^2^ of 0.9999 when comparing the output voltage with the input acceleration for the X- and Y-axes of the sensor. We developed an analytical, non-iterative, 12-parameter matrix calibration approach based on the least-squares method, which allows compensation for different gains in its axes, offset, and cross-axis. To improve the accuracy of our sensor, we propose a table with correction terms that can be subtracted from the estimated acceleration. The mean error of each estimated acceleration component of the sensor is zero, with a maximum standard deviation of 0.018 m/s^2^. The maximum RMSE for all tested positions is 6.7 × 10^−3^ m/s^2^.

## 1. Introduction

In this work, we focus on the full characterization of the accelerometer developed in [1] for seismic applications, which is a fiber Bragg grating (FBG)-based triaxial accelerometer that uses the overlapping interrogation method for the demodulation of the Bragg wavelength of the gratings. The common approach is to use two FBGs for each sensing axis; however, we require just one FBG for each sensor axis and one reference FBG. Thus, this device only needs four FBGs in total.

The relevance of accelerometers in the field of structural health monitoring lies in the fact that they are part of a system that measures different vibration parameters, such as strain, where FBGs can be directly embedded in buildings, as studied in [2]. Although acceleration can be calculated from displacement or velocity measurements and vice versa, the derivation or integration process causes large errors; thus, the direct measurement of the desired magnitude is preferred. Due to their good response, many researchers are working on the development of triaxial FBG-based accelerometers; for example, in [3], the authors developed a triaxial device with a measurement range between 0.1 Hz and 20 Hz and a high sensitivity of 606.2 pm/g; in [4], a triaxial sensor with an operating frequency between 0.1 Hz and 50 Hz was developed, achieving a sensitivity between 395 pm/g and 590 pm/g on its axes, and with a minimum detectable acceleration of 0.01 g; in both [3,4], the authors presented applications to seismic detection, similar to [5,6,7] and many other authors. These devices, and almost all FBG-based accelerometers, use an FBG demodulator (FBG interrogator) to measure the changes in the FBG. This FBG interrogator turns the sensor into a more complex and expensive device. We found that there is a lack of research into FBG sensors that do not use interrogators.

For the work presented here, we developed a new triaxial sensor assembled with new fiber Bragg gratings. Manufacturing helps to improve the orthogonality in its axes. This new sensor is based on the same operation principle, in which we do not use an interrogator to demodulate the FBG changes and use only one FBG for each axis and one additional reference FBG for all the axes. This approach was already employed, with satisfactory results, in the biaxial accelerometer developed in [8].

The characterization we perform in this work is of vital importance because it allows us to prove that our new device is suitable for the proposed application, and ensures that its measurements are reliable. Calibration methods are classified as static and dynamic characterizations. We selected a static characterization, which is performed by leaving the accelerometer fixed in different positions for a short period of time and recording the sensor output for each position; this procedure is the fundamental first step in the characterization of an accelerometer, since it allows us to determine the non-orthogonalities, the sensitivity in each axis, and the effects of temperature on the measurements.

Static calibration can be performed by iterative or analytical methods. Iterative methods have the advantage of not requiring one know the orientation of the accelerometer, as is the case with Newton’s method [9], the Levenberg–Marquardt algorithm, or the Thin-Shell algorithm [10], which try to minimize the calibration error in each iteration until it falls to a value equal to or smaller than a previously established one. In analytical methods, there is no need for iterations; however, the tilt angles of the accelerometer for each position must be known.

The analytical method was chosen in this work, which allows the linearity of the sensor to be determined in each of its axes by considering that its measurement range is less than ±0.5 g (±4.89 m/s^2^ in Mexico City), which can be provided by a tilt table [11]. The principle of this static calibration method is that the sum of three acceleration components in the accelerometer should be equal to the gravitational field vector [12].

On the other hand, since Bragg gratings are sensitive to both temperature and strain [13], we need to decouple the strain from temperature. In theory, the overlapping interrogation method of FBGs allows us to intrinsically compensate for temperature because the reference FBG does not undergo any change due to vibrations, only due to temperature changes; then, when each of the spectra of the sensing FBGs overlap with the spectrum of the reference FBG, the changes in the FBGs due to temperature are compensated. In practice, this compensation is not complete; in our prototype it is between 50% and 60%.

This incomplete compensation behavior has also been reported in other works, such as [14], where two FBGs that should have had the same sensitivity to temperature showed that they had different sensitivities in experiments: 23 pm/°C in one and 28 pm/°C in the other. Another example is [15], where the temperature coefficients of the two FBGs were 16.9 pm/°C and 18.2 pm/°C; thus, although the temperature dependence was reduced to 1.3 pm/°C, there was still some temperature dependence.

This imperfect temperature compensation approach has limited effects on the measurement of vibrations due to seismic motion, since the signal variation with respect to temperature is very small compared to the signal variations due to vibration. The longest period of the signal that our sensor is intended to measure is ten seconds; thus, the temperature variations are negligible, since they normally have a period of 24 h when the sensor is in a static position. Therefore, we can decouple the vibration signals from the temperature signals.

## 2. Materials and Methods

**Figure 1:**
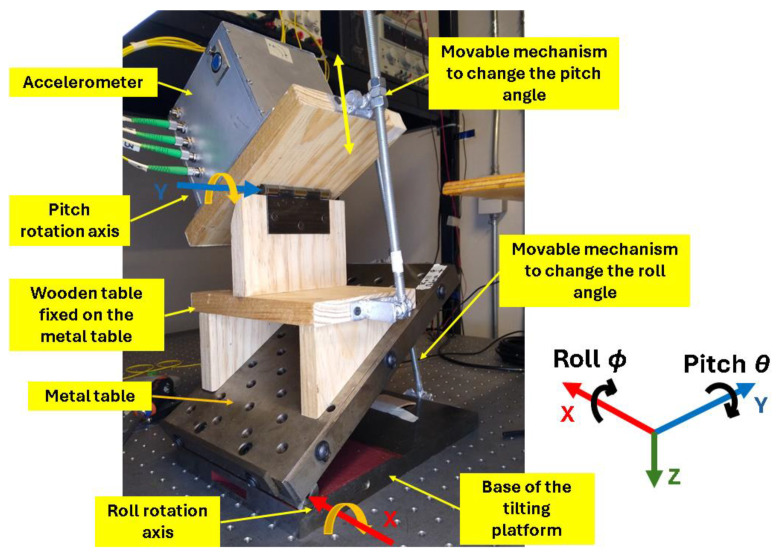
Tilting platform in pitch and roll rotations. The optical sensor is placed on top of the platform. The coordinate system is shown on the right of the figure.

The static characterization was performed on the hand-made tilting platform shown in Figure 1, whose parameters can be seen in Table 1. This platform allows us to change the pitch and roll angle by rotating the corresponding bolt nuts to slide one end of the tilting table. The hinges are tightened in such a way that there is no gap between their parts, allowing only rotational movement.

The magnitude of the gravitational acceleration varies with latitude, altitude, gravity anomaly, and tidal effect. We use the International Gravity Formula derived in [9], which depends on latitude and altitude, as shown in Equation (1).

(1) $$g\left(\gamma ,h\right)=9.780327\left[1+5.3024\times 10^{-3}\sin^{2}\left(\gamma \right)-5.08\times 10^{-6}\sin^{2}\left(\gamma \right)\right]-\;3.086\times 10^{-6}h\;\;[m/s^{2}],$$

where *γ* is the latitude, and *h* is the height (in meters) with respect to sea level. Substituting *γ* = +19.32078°, which corresponds to Mexico City’s latitude, and *h* = 2293 m according to [16], results in a local acceleration value of 9.7789 m/s^2^; this value will be used in the rest of the article as the magnitude of gravity.

As our accelerometer has a high sensitivity and a low range (±4.89 m/s^2^), it is enough to rotate its horizontal axes between a range of ±30 degrees. In the case of the vertical axis, to change the Z component of the gravity vector by 4.89 m/s^2^, it is necessary to tilt the platform within a ±60 degrees range. Therefore, the characteristics of the tilting platform are sufficient to characterize our sensor. The tilt angle of the platform was measured with a digital protractor model Pro 360 950-315 from the Japanese manufacturer Mitutoyo, which has a tilt range of 360 degrees and a resolution of 0.1 degree.

When the accelerometer is at rest in the horizontal position, the acceleration on the vertical axis of the sensor is 9.78 m/s^2^ downward, but since we want to measure the acceleration relative to the gravitational field, we take the acceleration as 0 m/s^2^ in all three axes at rest position. Another thing to note in the vertical axis is that the changes in acceleration are symmetric regardless of whether the platform is tilted in the positive or negative sense. Thus, while the horizontal axes are tested over a range of ±4.89 m/s^2^, the vertical axis is only tested from 0 to −4.89 m/s^2^. The accelerometer cannot be turned over (rotated 180 degrees about the X- or Y-axis) because it is outside the measurement range of the sensor.

### Static Calibration Procedure

The most general linear calibration of the accelerometer has a total of 12 parameters [17], where a recalibration is performed over the previous factory calibration. We use this same procedure to perform the first calibration of our sensor. This is a non-iterative method, which is based on the linear least-squares optimization.

At first, the general linear model that relates the dependent variable *y* to the *N* inputs $x_{0}$ to $x_{N-1}$, through *N* coefficients $\beta_{0}$ to $\beta_{N-1}$, is

(2) $$y=\beta_{0}x_{0}+\beta_{1}x_{1}+\beta_{2}x_{2}+\ldots +\beta_{N-1}x_{N-1}.$$

The *N* model parameters for each sensor axis $\beta_{0}$ to $\beta_{N-1}$ are determined from the *M* measurements from *i* = 0 to *i* = *M* − 1, where *M* ≥ *N*. Equation (2), written in vector form for all *M* measurements, is

(3) $$\left(\begin{array}{c} y\left[0\right]\\ y\left[1\right]\\ \vdots \\ y\left[M-1\right] \end{array}\right)=\left(\begin{array}{cccc} x_{0}\left[0\right] & x_{1}\left[0\right] & \cdots & x_{N-1}\left[0\right]\\ x_{0}\left[1\right] & x_{1}\left[1\right] & \cdots & x_{N-1}\left[1\right]\\ \vdots & \vdots & \ddots & \vdots \\ x_{0}\left[M-1\right] & x_{1}\left[M-1\right] & \cdots & x_{N-1}\left[M-1\right] \end{array}\right)\left(\begin{array}{c} \beta_{0}\\ \beta_{1}\\ \vdots \\ \beta_{N-1} \end{array}\right).$$

The vector ***Y*** of the dependent variables for each sensor axis is defined as

(4) $$Y=\left(\begin{array}{c} y\left[0\right]\\ y\left[1\right]\\ \vdots \\ y\left[M-1\right] \end{array}\right).$$

The matrix ***X*** of the measurements of the independent variables is defined as

(5) $$X=\left(\begin{array}{cccc} x_{0}\left[0\right] & x_{1}\left[0\right] & \cdots & x_{N-1}\left[0\right]\\ x_{0}\left[1\right] & x_{1}\left[1\right] & \cdots & x_{N-1}\left[1\right]\\ \vdots & \vdots & \ddots & \vdots \\ x_{0}\left[M-1\right] & x_{1}\left[M-1\right] & \cdots & x_{N-1}\left[M-1\right] \end{array}\right).$$

The solution vector *β* of the *N* model parameters for each sensor axis is defined as

(6) $$\beta =\left(\begin{array}{c} \beta_{0}\\ \beta_{1}\\ \vdots \\ \beta_{N-1} \end{array}\right).$$

Using Equations (4)–(6), Equation (3) can be rewritten as

(7) Y=Xβ.

In the linear least-squares optimization, a residuals vector is defined as

(8) r=Y−Xβ.

The optimal least-squares fit for *β* is the one that minimizes the performance function *P* defined as the squared modulus of the residuals vector [17]. Finally, the commonly known normal equations for least squares optimization result in:

(9) $$\beta ={\left(X^{T}X\right)}^{-1}X^{T}Y.$$

The matrix ***X*** of measurements is reused in the calculation of the parameter of the three axes; the vectors ***Y*** and *β* are different for each axis. The calibrated accelerometer output ***G*** is defined by

(10) $$G=\left(\begin{array}{c} G_{x}\\ G_{y}\\ G_{z} \end{array}\right)=WV_{0}+V=\left(\begin{array}{ccc} W_{xx} & W_{xy} & W_{xz}\\ W_{yx} & W_{yy} & W_{yz}\\ W_{zx} & W_{zy} & W_{zz} \end{array}\right)\left(\begin{array}{c} V_{ox}\\ V_{oy}\\ V_{oz} \end{array}\right)+\left(\begin{array}{c} V_{x}\\ V_{y}\\ V_{z} \end{array}\right),$$

where ***W*** is the gain matrix, which has nine independent elements. It includes all possible cross-axis interactions and any internal rotation of the sensor structure with respect to its case. ***V***_0_ are the output voltages of the sensor, and ***V*** is the offset vector at 0 m/s^2^. Using Equation (9), the optimum least squares solution for $\beta_{x}$ of the X-axis is

(11) $$\beta_{x}=\left(\begin{array}{c} W_{xx}\\ W_{xy}\\ W_{xz}\\ V_{x} \end{array}\right)={\left(X^{T}X\right)}^{-1}X^{T}Y_{x}={\left(X^{T}X\right)}^{-1}X^{T}\left(\begin{array}{c} -\sin\theta \left[0\right]\\ -\sin\theta \left[1\right]\\ \vdots \\ -\sin\theta \left[M-1\right] \end{array}\right).$$

Similarly, the optimum least-squares solution for the remaining eight calibration parameters for the Y- and Z-axes is

(12) $$\beta_{y}=\left(\begin{array}{c} W_{yx}\\ W_{yy}\\ W_{yz}\\ V_{y} \end{array}\right)={\left(X^{T}X\right)}^{-1}X^{T}Y_{y}={\left(X^{T}X\right)}^{-1}X^{T}\left(\begin{array}{c} \cos\theta \left[0\right]\sin\phi \left[0\right]\\ \cos\theta \left[1\right]\sin\phi \left[1\right]\\ \vdots \\ \cos\theta \left[M-1\right]\sin\phi \left[M-1\right] \end{array}\right),$$

(13) $$\beta_{z}=\left(\begin{array}{c} W_{zx}\\ W_{zy}\\ W_{zz}\\ V_{z} \end{array}\right)={\left(X^{T}X\right)}^{-1}X^{T}Y_{z}={\left(X^{T}X\right)}^{-1}X^{T}\left(\begin{array}{c} \cos\theta \left[0\right]\cos\phi \left[0\right]\\ \cos\theta \left[1\right]\cos\phi \left[1\right]\\ \vdots \\ \cos\theta \left[M-1\right]\cos\phi \left[M-1\right] \end{array}\right).$$

The measurement matrix ***X*** is common to all three Equations (11)–(13). The angle *θ* is the pitch rotation about the Y-axis, the angle *ϕ* is the roll rotation about the X-axis, and *M* is the number of different sensor orientations used to find the calibration parameters.

## 3. Results

In this section, we show the dependence of the Bragg wavelength of the four FBGs used in the sensor on temperature changes, and how to compensate for them. We show that the reference FBG is not affected by vibrations, only by temperature. The response of our sensor to input acceleration, in wavelength and voltage, is shown, along with the 12-parameter model calibration and error analysis.

### 3.1. Bragg Wavelength Dependence of FBGs with Temperature

As mentioned in the introduction, the reference FBG acts as a temperature compensation device, which in theory must be 100%. In practice, all FBGs have different sensitivity coefficients to a greater or lesser degree. To characterize the dependence on temperature changes in all four FBGs used in our sensor, we kept the sensor stable at a fixed position and changed the temperature of the sensor. We placed an incandescent lamp over the sensor at a certain distance from it, which was periodically reduced to increase the sensor temperature. The internal temperature of the sensor, measured using a DHT22 sensor, was changed from 24 °C to 31 °C. The Bragg wavelength was recorded with a device, the Micron Optics—Optical Sensing Interrogator sm130, with a sampling rate of 100 Hz. To reduce noise, 1000 samples were averaged for each data point [18]. Although the external temperature was changed from 24 °C to about 50 °C, the internal temperature did not reach the same value, which means that the sensor case helps to reduce the variation of the output voltages due to temperature. The total time to change the temperature from 24 °C to 31 °C was 3 h and 25 min.

**Figure 2:**
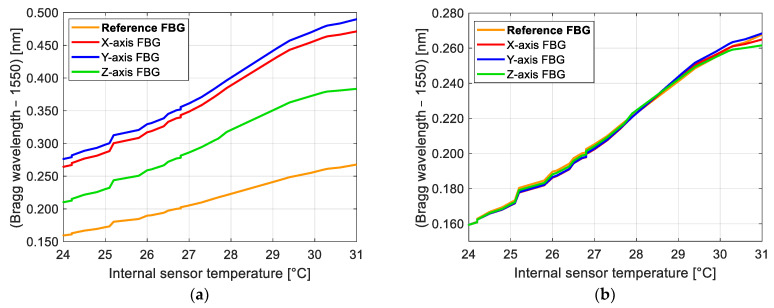
Dependence of the FBGs of our prototype on temperature: (**a**) apart from an offset, the sensitivity in all FBGs is different; (**b**) the Bragg wavelength of sensing FBGs is adjusted by a factor and an offset; now all FBGs show the same trend.

Figure 2a shows the Bragg wavelengths of all FBGs used in our sensor with respect to its internal temperature. To compare the responses of the gratings, they must have the same initial Bragg wavelength; therefore, an offset was applied to the Bragg wavelength of the sensing FBGs. Then, the Bragg wavelength of the sensing FBGs was adjusted by a factor using Equation (14), which changes the temperature sensitivity. The reference FBG has the lowest sensitivity to temperature; thus, the adjustment factors found for the sensing gratings were less than 1—0.51, 0.51, and 0.59 for the X-, Y-, and Z-axes, respectively. The temperature adjustment is then of the form

(14) $$\lambda_{BS_{adj},i}=\lambda_{BS,0}+\left(\lambda_{BS,i}-\lambda_{BS,0}\right)f_{a}+O$$

where $\lambda_{BS_{adj},i}$ is the *i*-th adjusted Bragg wavelength of the *i*-th measurement point, $\lambda_{BS,0}$ is the Bragg wavelength of the first measurement point, $\lambda_{BS,i}$ is the Bragg wavelength of the *i*-th measurement point, $f_{a}$ is the adjustment factor, and *O* is the offset. Equation (14) is applied for each of the sensing axes.

**Figure 3:**
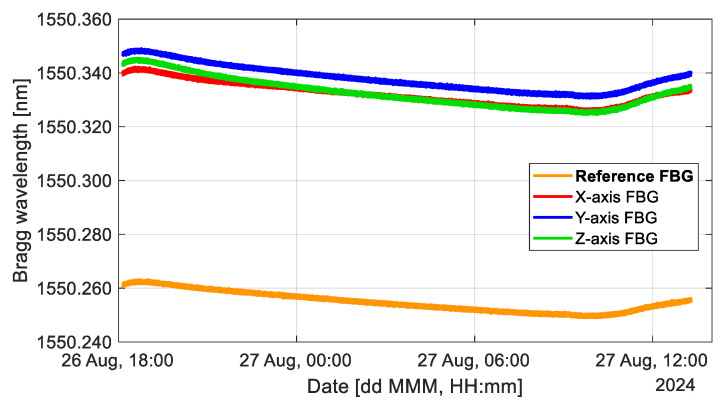
Changes in Bragg wavelength due to temperature over 17 h at laboratory room temperature. Measurements were made with a sampling period of 0.1 s, and a simple moving average with a window size of 5 was applied to the data.

In Figure 3, we can see the responses of the FBGs of our sensor, which was left recording in the laboratory for 17 h. The accelerometer was left on an optical table with active pneumatic isolation; thus, it would not suffer from the ambient vibrations of the building. The changes in the Bragg wavelength of the FBGs are due to temperature, in the same way as in Figure 2. This figure was added since the accelerometer is not intended to work at a controlled ambient temperature, and the temperature surrounding it will change in a similar way as in Figure 3. The trend in this figure confirms once again that the Bragg wavelength of all FBGs shows the same tendency with time, as the ambient temperature changes. The initial Bragg wavelength of the FBGs may vary depending on the sensor calibration.

Therefore, we can state that the reference FBG, when used in the overlapping interrogation method, helps to decrease the influence of temperature by reducing the drift of the sensor output. By using the adjustment factors to change the sensitivity of sensing FBGs, we can almost eliminate the signal variation. In addition, the signal variation due to temperature is very small compared to the variations due to vibrations. This is because the temperature changes at a very slow rate compared to the period of vibrations.

### 3.2. Inmunity of Reference FBG to Vibrations

**Figure 4:**
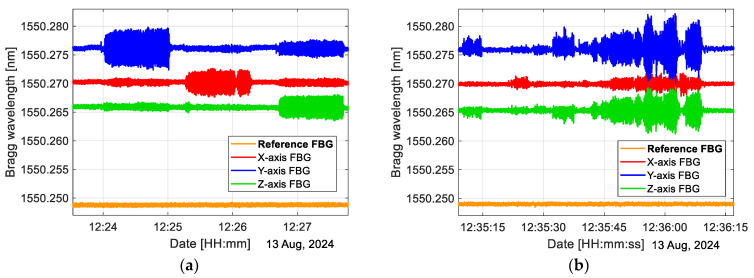
Vibration is applied to the tilting platform to which our sensor is attached; it can be noted that the reference FBG is not affected by such vibration: (**a**) vibration applied in a preferred direction; the X-, Y-, or Z-axis; (**b**) vibration applied in arbitrary directions.

The reference FBG has two purposes in our sensor: the first is to complete the demodulation scheme for the accelerometer, and the second is to compensate for temperature. Therefore, it is very important that the reference FBG is not affected by vibrations. In our prototype sensor, this fiber grating is fixed in an aluminum clamp with a set screw for pretension [1]. This grating is not connected to the inertial mass as the sensing gratings are; thus, this FBG is not affected by vibrations as demonstrated in Figure 4. In Figure 4a, we apply a directional vibration by hitting the tilting platform to which the accelerometer is attached. It can be seen that the reference FBG is not affected by these hits. In Figure 4b, we apply aleatory shocks, and we can observe that all three axes are affected, but, again, the reference FBG is not affected by these impulses.

We performed a simple moving average with a window size of 10 on data in Figure 4 that were sampled at 1 kHz.

The reference FBG is attached to the sensor structure by two points with a 25 mm clearance between the fixing points; this clearance is the same as that of the sensing grating; therefore, the immunity of the reference FBG to vibrations is proof that the possible bending of optical fibers under the influence of acceleration is decreased by the pre-strain applied to the fiber at the time of its fixing, as reported in [19]. Although the pre-strain limits the maximum measurable acceleration, since in our case the maximum acceleration to be measured is only ±4.89 m/s^2^, this limitation does not affect it. Due to the pre-strain, the fiber is never slack, but its tension is higher or lower depending on the direction of motion; therefore, the bending of the fiber is decreased, and its effect is not seen in the result of the experiment.

### 3.3. Static Characterization of the Accelerometer

#### 3.3.1. Wavelength Characterization

As described in the materials and methods section, the X-axis was tilted in a ±60 degrees range to obtain the response of the vertical axis to −0.5 g, while the Y-axis was only tilted in a ±30 degrees range. The step in the measurements was 5 degrees.

**Figure 5:**
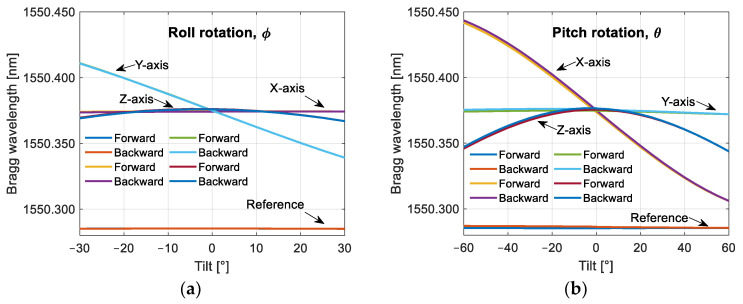
Response in wavelength of the FBGs used in our prototype with respect to tilt. The tilt is referenced to the horizontal plane; two sweeps were performed: one forward and one backward. (**a**) The sensor is rotated about its X-axis; (**b**) the sensor is rotated about its Y-axis.

**Figure 6:**
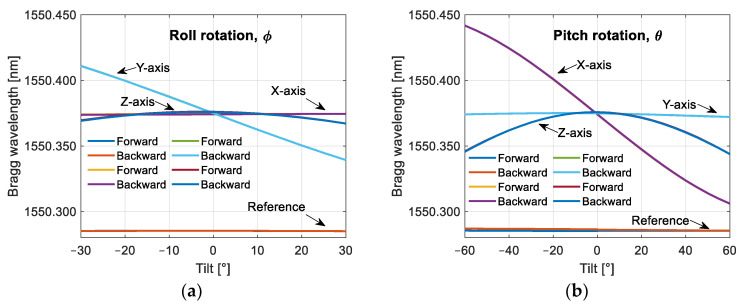
Response in wavelength of the FBGs used in our prototype with temperature compensation. The tilt is referenced to the horizontal plane; the temperature compensation was performed with respect to the reference FBG: (**a**) the sensor is rotated about its X-axis; (**b**) the sensor is rotated about its Y-axis.

In Figure 5, we can see a little offset between the forward and the backward sweep measurements due to temperature changes; this offset is clearer in Figure 5b. Before analyzing this data, we performed compensation based on the changes of the reference FBG using Equation (15).

(15) $$\lambda_{BS_{adj},i}=\lambda_{BS,i}-\left(\lambda_{BR,i}-\lambda_{BR,0}\right)f_{ar},$$

where $\lambda_{BS_{adj},i}$ is the *i*-th adjusted Bragg wavelength of the *i*-th measurement point, $\lambda_{BS,i}$ is the Bragg wavelength of the *i*-th measurement point, $\lambda_{BR,i}$ is the Bragg wavelength of the *i*-th measurement point of reference FBG, $\lambda_{BR,0}$ is the Bragg wavelength of the initial measurement point of the reference FBG, and $f_{ar}$ is the adjustment factor. Equation (15) applies to each of the sensing axes. The adjustment factors used in the compensation were 1.15, 0.90, and 1.00 for the X-, Y-, and Z-axes, respectively. The results are shown in Figure 6, where the response of reference FBG is shown without compensation.

**Figure 7:**
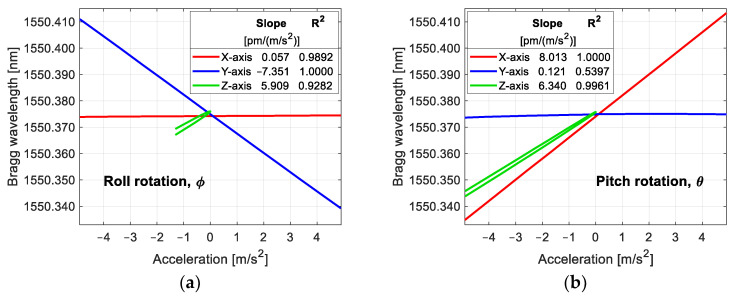
Response of the sensing FBG of our sensor, the horizontal axis of the plot was converted to acceleration by using the sine function of the tilt angle for the X- and Y-axes and the cosine function for the Z-axis. In the Z-axis, the magnitude of the gravity vector is subtracted from the measurements: (**a**) the sensor is rotated about its X-axis; (**b**) the sensor is rotated about its Y-axis.

After temperature compensation, the forward and backward sweep data are averaged for each sensor orientation and plotted with the horizontal axis in units of acceleration. The acceleration was calculated with Equation (16) by applying the sine function to the tilt angles in the X- and Y-axes and the cosine function for the vertical axis of the sensor. The results are shown in Figure 7.

(16) $$a_{x}=-\sin\theta \;a_{y}=\cos\theta \sin\phi \;a_{z}=\cos\theta \cos\phi$$

where $a_{x}$, $a_{y}$, and $a_{z}$ are the components of the gravity vector coupled to the X-, Y-, and Z-axes of the sensor, *θ* is the pitch rotation about the Y-axis, and *ϕ* is the roll rotation about the X-axis of the sensor.

For Figure 7a, where the sensor rotates about its X-axis, after a linear fit, we obtained a sensitivity of −7.351 pm/(m/s^2^) for the Y-axis with an R-squared (correlation factor) of 1.0000, while we have a cross-sensitivity of 0.057 pm/(m/s^2^) on the X-axis. This means a cross-sensitivity 129 times lower than the corresponding axis sensitivity. In the second case, where the sensor is rotated about its Y-axis, we obtained a sensitivity of 8.013 pm/(m/s^2^) for the X-axis with an R-squared value of 1.0000, and 0.121 pm/(m/s^2^) on the Y-axis; thus, we have a cross-sensitivity 66 times lower than the corresponding axis.

For the Z-axis, where the acceleration is symmetrical with respect to both senses of rotation of the horizontal axes of the sensor, the ideal would be to obtain a line that goes from −5 to 0 m/s^2^, then from 0 to −5 m/s^2^, arriving at the same place; however, in Figure 7a,b, we can see that its sensitivity is somewhat different depending on the sense of rotation of the sensor. If we analyze Figure 6, the difference in sensitivity can be attributed to a slight misalignment of the Z-axis with respect to the vertical. This is because the maximum wavelength of the Z-axis FBG is not obtained when the sensor is in a flat position, but when it is somewhat tilted. The average sensitivity of this axis, when the sensor is rotated about the X-axis, is 5.909 pm/(m/s^2^), with R-squared equal to 0.9282, and 6.340 pm/(m/s^2^) when the sensor is rotated about the Y-axis, with R-squared equal to 0.9961.

#### 3.3.2. Voltage Characterization

**Figure 8:**
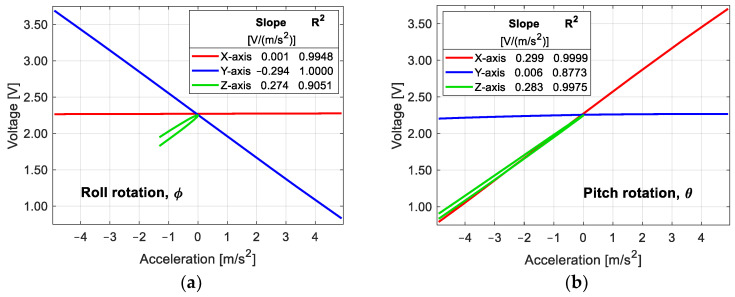
Sensor output in voltage at the final sensor stage with respect to the input acceleration: (**a**) rotation about the X-axis; (**b**) rotation about the Y-axis.

The sensitivities and response curves we obtained earlier were at the wavelength scale with respect to the acceleration experienced in each axis. These values help to reveal the response of our sensor to temperature and acceleration during characterization. However, one of the main advantages of our sensor is that we do not need interrogation equipment; instead, the reference FBG allows us to demodulate the Bragg wavelength of the sensing fiber gratings into optical power changes, which are measured with simple photodetectors. The output of the photodetectors is a current, which is proportional to the input optical power. Hence, the next step is to obtain the response of our sensor by measuring the voltage at the output of the electronic stage. The way of characterization is the same as in the wavelength characterization; the sweeps are in the same range, and the results are shown in Figure 8.

According to Figure 8a, the sensitivity of the Y-axis when the sensor rotates about the X-axis (roll rotation) is −0.294 V/(m/s^2^) with a correlation coefficient of 1.0000, and 0.001 V/(m/s^2^) for the X-axis, which means a cross-sensitivity 267 times lower (0.34%) than that of the corresponding Y-axis. When the sensor rotates about the Y-axis (pitch rotation), the sensitivity for the X-axis is 0.299 V/(m/s^2^) with R-squared equal to 0.9999, and a cross-sensitivity of 0.006 V/(m/s^2^) for the Y-axis, i.e., a cross-sensitivity 48 times lower (2.08%) than that of the X-axis. For the Z-axis, the average sensitivity when the sensor rotates about the X-axis is 0.274 V/(m/s^2^) and 0.283 V/(m/s^2^) when it rotates about the Y-axis. The cross-axis interference for our sensor is comparable to that of some commercial accelerometers, such as the SF3000L calibrated in [12].

The overlapping interrogation method has a nonlinear response because the output power of the method is a convolution of the two FBG spectra, modeled as Gaussians [20]. There is a region where the response of this method is nearly linear; this region can be achieved with a suitable separation between the Bragg wavelength of the reference FBG and that of the sensing FBG. The correlation coefficient that we found in the sensor for the X- and Y-axes is very close to unity, which means that the FBGs are working in the linear region. The proper separation between the reference FBG and the sensing FBGs found experimentally in our sensor is shown in Table 2.

#### 3.3.3. The 12-Parameters Calibration Model

Along with the voltage characterization, we performed the calibration model described in Equation (10). The sensor orientations used as input for parameter estimation are shown in Table 3, where *θ* is the angle of rotation about the Y-axis of the sensor from the horizontal plane, and *ϕ* is the angle of rotation about the X-axis of the sensor from the horizontal plane too. For the estimation of the X- and Y-axes parameters, the positions 0, 1, 2, 4, 5, and 6 were used. For the Z-axis, all eight positions were used.

The 12-parameter model includes three main gains, cross-axis gains, sensor axes misalignment, and offsets for zero input. The model parameters’ values found for our sensor are shown in Equation (17).

(17) $$G=\left(\begin{array}{c} G_{x}\\ G_{y}\\ G_{z} \end{array}\right)=\left(\begin{array}{ccc} \;\;\;\;3.3610 & \;\;\;0.0171 & -0.1107\\ \;\;\;0.0719 & -3.4242 & \;\;\;0.0844\\ -0.0608 & -0.1500 & \;\;\;3.5454 \end{array}\right)\left(\begin{array}{c} V_{ox}\\ V_{oy}\\ V_{oz} \end{array}\right)+\left(\begin{array}{c} -7.4219\\ \;\;\;7.3733\\ -7.5029 \end{array}\right).$$

In the estimated parameters, the vertical acceleration is considered relative to the magnitude of the gravity field; then, at the rest position of the sensor, all three axes have a zero value. $G_{x}$, $G_{y}$, and $G_{z}$ are the acceleration components in m/s^2^ for the X-, Y-, and Z-axes, respectively. $V_{ox}$, $V_{oy}$, and $V_{oz}$ are the output voltages of the accelerometer electronic stage in volts.

#### 3.3.4. Errors in the 12-Parameter Calibration Model

We verified the estimated calibration model parameters for our sensor by introducing the sensor output voltages from each of the sensor orientations obtained during the sweeps, as shown in Figure 5, and comparing the model output to each acceleration component exerted at each position. We calculate the full-scale error (FSE) as a percentage for all measurements with Equation (18).

(18) $$FSE\;\left[\%\right]=\frac{measured\;value-reference\;value}{full-scale\;aceleration}\times 100\%.$$

The maximum full-scale errors are 0.58%, 0.48%, and 0.39% for the X-, Y-, and Z-axes, respectively. In absolute values, the maximum acceleration errors were 0.057 m/s^2^, 0.047 m/s^2^ and 0.038 m/s^2^ for the X-, Y-, and Z-axes, respectively. These errors found are comparable to the errors found after calibration in [12].

In the estimation of the calibration parameters, the 38 recorded positions were also considered to see how much the maximum error decreased, finding that there was no improvement. Therefore, we can conclude that using eight orientations to estimate the model parameters is a good compromise between the number of positions and the error obtained.

**Figure 9:**
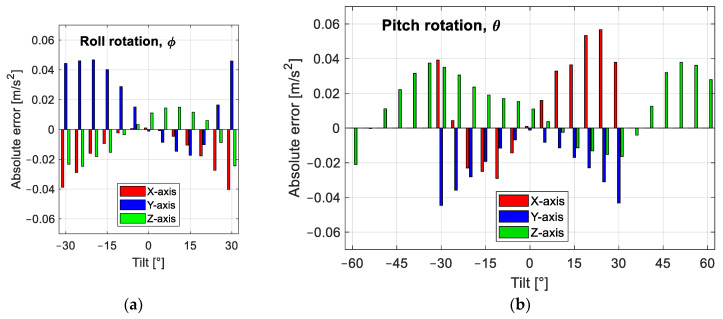
Absolute acceleration error comparing the theoretical acceleration due to each tilt angle with the estimated acceleration using the sensor output voltages: (**a**) the sensor is rotated about its X-axis; (**b**) the sensor is rotated about its Y-axis.

In Figure 9, we show the errors present after calibration for each sensor orientation in each axis. The largest errors are present at the extremes of the sensor range, i.e., near ±4.89 m/s^2^. The plot has the horizontal axis in units of angles because this unit is common to the horizontal and vertical axes of the sensor, but acceleration is not. The errors are not totally random, as they show a tendency to increase or decrease systematically.

**Figure 10:**
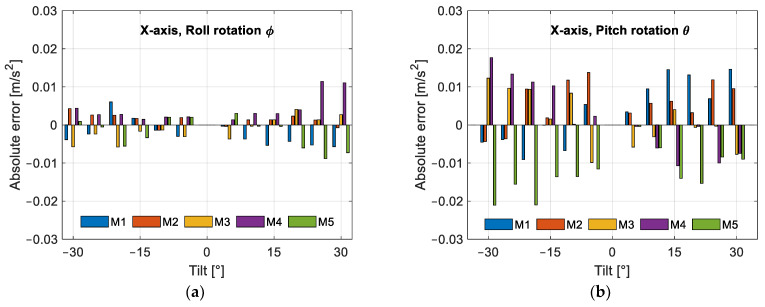

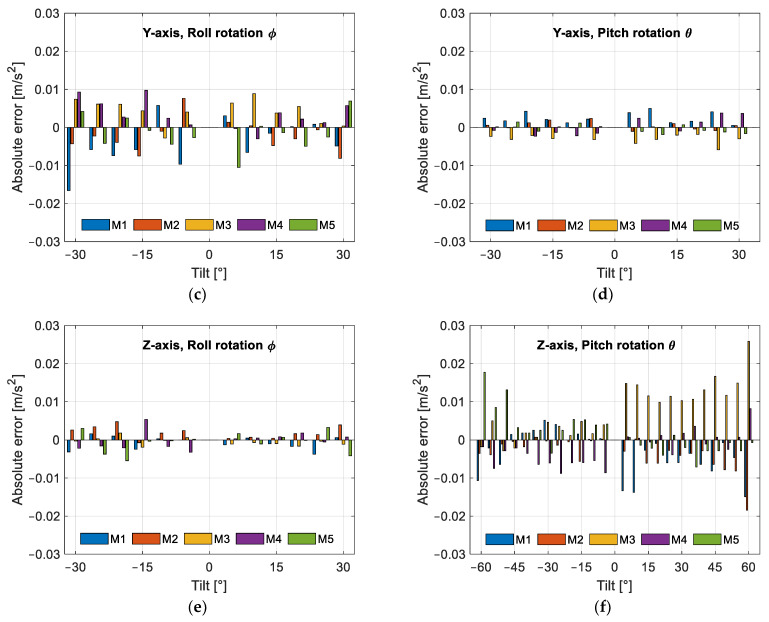
Absolute acceleration error after subtracting the mean error for each sensor orientation: (**a**) X-axis response to roll rotation; (**b**) X-axis response to pitch rotation; (**c**) Y-axis response to roll rotation; (**d**) Y-axis response to pitch rotation; (**e**) Z-axis response to roll rotation; (**f**) Z-axis response to pitch rotation.

To further reduce errors, we propose to subtract one term from each estimated component of acceleration. To make sure our proposal is adequate and that the errors decrease, we performed the sweep for the 38 positions five times. We then subtracted the mean error of the five measurements at each position. In Table 4, we can see the terms to be subtracted from the estimated acceleration together with the standard deviation of the error at each orientation. The remaining errors are plotted in Figure 10, where we group the errors for each position and each axis. Now, the errors have a zero mean and show a random behavior.

From Figure 10a,d,e, we see that the errors are below ±0.01 m/s^2^, while in Figure 10b,c, the maximum errors are around ±0.02 m/s^2^, with the largest maximum error in Figure 10f being between +0.03 and −0.02 m/s^2^. With the correction term applied to the estimated acceleration components, the maximum errors for all sweeps are as follows: for the X-axis ±0.02 m/s^2^, for the Y-axis between +0.01 and −0.02 m/s^2^, and for the Z-axis between +0.03, and −0.02 m/s^2^. In percentage, the maximum error was reduced by 58%, 58%, and 54% for the X-, Y-, and Z-axes, respectively.

**Figure 11:**
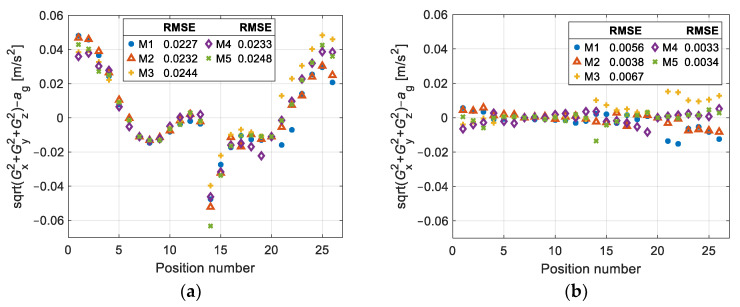
Absolute acceleration error, between the magnitude of the calibrated output of the accelerometer and the magnitude of the gravity field, and their respective RMSEs: (**a**) errors before subtracting the correction term; (**b**) errors after subtracting the correction term.

Figure 11 shows the error between the gravity magnitude of the calibrated accelerometer output and the magnitude of the gravity field. Figure 11a shows the errors in each sensor orientation for each of the five sweeps and the root mean square error (RMSE), with the maximum value being 0.025 m/s^2^ before error correction. From Figure 11b, we can see that the RMSE decreases to 0.007 m/s^2^; therefore, the RMSE is reduced by 72%. In Table 5, we can see the pitch and roll angle for each of the positions in Figure 11.

**Figure 12:**
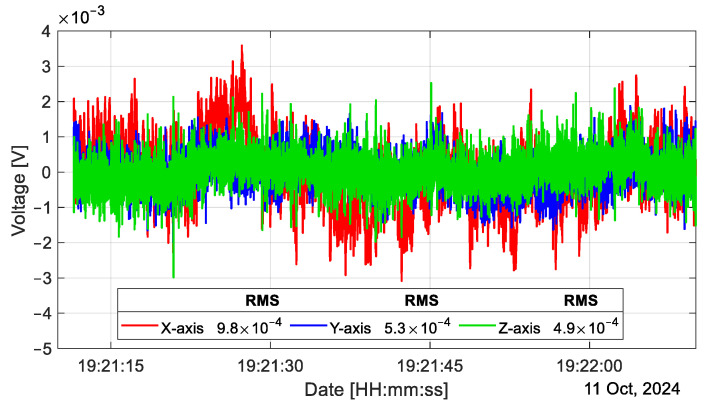
Electrical noise present at the output of the accelerometer electronic stage in a 60 s window.

Now, we compare the errors obtained during calibration and the noise present in the circuit. The root mean square (RMS) values of the noise, which we can see in Figure 12, are 9.8 × 10^−4^ V, 5.3 × 10^−4^ V, and 4.9 × 10^−4^ V for the X-, Y-, and Z-axes, respectively. These values are converted to acceleration using the sensitivities shown in Figure 8, and result in 0.0033 m/s^2^, 0.0018 m/s^2^ and 0.0017 m/s^2^, for the X-, Y- and Z-axes, respectively. The cutoff frequency is 20 Hz, and the window size is 58 s. The noise we see in Figure 12 is the sum of all noise sources present in the electronic circuit, which includes the thermal noise within the analog-to-digital converter and quantization noise due to the analog-to-digital conversion process [21]. The SNR (signal-to-noise ratio) is calculated using

(19) $$SNR=20\log\left(\frac{full-scale\;input}{noise}\;\right).$$

The full-scale input of our sensor is 9.7789 m/s^2^, which, when converted to RMS, is 3.4574 m/s^2^. The full-scale input (or range) is converted to RMS for consistency with the RMS noise used in the calculation. Using Equation (19), the SNR values for the X-, Y-, and Z-axes are 60.40 dB, 65.7 dB, and 66.2 dB, respectively. On the other hand, the dynamic range (DR) is defined as

(20) $$DR=20\log\left(\frac{range}{2\times noise}\;\right).$$

DR is found to be 54.4 dB, 59.6 dB, and 60.1 dB for the X-, Y-, and Z-axes, respectively. We consider the minimum detectable acceleration as twice the noise value, i.e., the minimum detectable acceleration is 6.6 × 10^−3^ m/s^2^, 3.6 × 10^−3^ m/s^2^, and 3.4 × 10^−3^ m/s^2^.

## 4. Discussion and Conclusions

The characterization of a new triaxial FBG-based accelerometer has been performed. This triaxial accelerometer uses only a total of four fiber gratings. This has been achieved by reusing the reference FBG, which is not part of any sensor axis, with each of the sensing axes. We need to confirm that the Bragg wavelength of the reference FBG is not affected by vibrations, and that we can reuse it for demodulating the Bragg wavelength of the three sensing axes without loss of linearity. Therefore, the first experiment was to apply acceleration on the sensor axes, where the results show that the reference FBG is immune to vibrations. The reference FBG is oriented along the X-axis of the sensor, but because it is vibration-insensitive, its orientation has no effect on the responses of the sensing FBGs.

We have shown that all FBGs exhibit similar behaviors with respect to temperature, and that although the temperature compensation in the sensing FBGs is not at 100%, the reference FBG helps to compensate between 50% and 60%. The remaining temperature dependence can be neglected because the signal variation due to temperature is much slower than the signal variation due to vibration. Over a short period of time, the signal variation is also very small.

The overlapping interrogation method is nonlinear; then, the linearity of the output power from the overlapping interrogation method with respect to the input acceleration is another issue. A nearly linear operating range can be used with a suitable Bragg wavelength difference between the sensing FBG and the reference grating. The optimal spacing for the FBGs used in our sensor was found to be 102 ± 2 pm, 98 ± 2 pm, and 95 ± 2 pm for the X-, Y-, and Z-axes, respectively. Since the correlation factors for the X- and Y-axes are 1.0000 and 0.9999, respectively, we are operating within the nearly linear operating range, and the linear model of the sensor could be constructed.

We chose an analytical calibration method instead of an iterative one in order to determine the linearity of our sensor and to make the necessary adjustments to the Bragg wavelength of the gratings until we were operating in the center of the linear range of the demodulation scheme. We performed a 12-parameter calibration based on the linear least squares optimization by placing the sensor in seven different positions. The full-scale errors found were all under 1%—0.58%, 0.48%, and 0.39% for the X-, Y-, and Z-axes, respectively. We performed five sweeps in all sensor orientations, and the errors in the estimated acceleration components were repetitive; thus, we proposed to subtract a term, equal to the average of the five sweeps, from the estimated acceleration. After error compensation, the values obtained were 0.24%, 0.20%, and 0.18% for the X-, Y-, and Z-axes, which means reductions of 58%, 58%, and 54%, respectively.

The dynamic range found in the sensor was 54.4 dB, 59.6 dB, and 60.1 dB for the X-, Y-, and Z-axes, respectively. The minimum detectable acceleration was 6.6 × 10^−3^ m/s^2^, 3.6 × 10^−3^ m/s^2^, and 3.4 × 10^−3^ m/s^2^ for the X-, Y-, and Z-axes, respectively. The minimum detectable acceleration was small enough to detect a seismic event of magnitude less than 3.0, and the errors in the measurements were all low enough to estimate the amplitude of the motions accurately. The larger cross-sensitivity value for our sensor was found to be 1.51%.

The characteristics of our sensor are comparable to those of the high-figure-of-merit, low-cross-sensitivity accelerometer developed in [3], where the minimum detectable acceleration was 1.67 × 10^−3^ m/s^2^ with a transverse sensitivity of 2.3%. Although the accelerometer in [3] showed a higher sensitivity, 606.2 pm/g, compared to 78.4 pm/g, 71.9 pm/g, and 57.8 pm/g for the X-, Y-, and Z- axes of our sensor, we did not have the limitation of the interrogator resolution, and this has allowed us to achieve similar minimum detectable acceleration. Our sensor improved by 15-fold the minimum detectable acceleration when compared to that in [4], the value of which was 9.8 × 10^−2^ m/s^2^. The low-frequency accelerometer developed in [22] had a sensitivity of 681.7 pm/g and a transverse interference of less than 4.9%, and, considering the errors in the sensor, the minimum acceleration amplitude that it could measure was around 2.9 × 10^−1^ m/s^2^. In [23], a high sensitivity of 763.2 pm/g was achieved, but again, the minimum detectable acceleration was limited by the resolution of the interrogator used, which in this case was 1 pm, meaning that its minimum detectable acceleration was about 1.3 × 10^−2^ m/s^2^, and its cross-sensitivity was 5%. It is worth mentioning that the accelerometers mentioned above used an interrogator. Therefore, it is demonstrated that our sensor, which does not need an interrogator and uses only one FBG for each sensing axis, shows similar or better characteristics than those that do.

Our accelerometer can be used to measure vibration response in buildings, and help preserve structures and human lives. This device does not require an interrogator and can be operated as an autonomous unit. As future work, since the sensor sensitivity may vary with frequency, a dynamic characterization of the sensor is needed. The reduction in its size will improve internal temperature uniformity and further reduce the possibility of optical fiber bending with vibration. Further, the construction of the sensor frame in one piece will prevent possible resonances after prolonged use. This developed accelerometer prototype is suitable for field applications, and is not only a laboratory experiment.

## Figures and Tables

**Table 1:** Characteristics of the tilting platform.

Parameter	Range	Resolution
Pitch rotation, *θ* (Rotation about the Y-axis)	±60 deg	0.1 deg
Roll rotation, *ϕ* (Rotation about the X-axis)	±30 deg	0.1 deg

**Table 2:** Optimal separation between the Bragg wavelength of sensing FBGs and the reference FBG.

FBG_1_	FBG_2_	Bragg Wavelength Separation (pm)
Reference	X-axis FBG	102 ± 2
Reference	Y-axis FBG	98 ± 2
Reference	Z-axis FBG	95 ± 2

**Table 3:** Orientations for sensor calibration.

Orientation Number	Pitch, θ [deg]	Roll, *ϕ* (deg)
0	0	−30
1	0	0
2	0	+30
3	−60	0
4	−30	0
5	0	0
6	+30	0
7	+60	0

**Table 4:** Correction terms to be subtracted from estimated accelerations and the respective standard deviation (SD) of the zero-mean error.

Ref. Angle θ; *ϕ* [deg]	Correction Term $C_{Gx}$$,\;C_{Gy}$$,\;C_{Gz}$ (m/s^2^)	Error SD $\sigma_{Ex}$$,\;\sigma_{Ey}$$,\;\sigma_{Ez}$ (m/s^2^)	Ref. Angle θ; *ϕ* (deg)	Correction Term $C_{Gx}$$,\;C_{Gy}$$,\;C_{Gz}$ (m/s^2^)	Error SD $\sigma_{Ex}$$,\;\sigma_{Ey}$$,\;\sigma_{Ez}$ (m/s^2^)
0; −30	0.0388; −0.0443; 0.0233	0.0047; 0.0106; 0.0027	−30; 0	−0.0391; 0.0446; −0.0351	0.0154; 0.0018; 0.0049
0; −25	0.0288; −0.0461; 0.0247	0.0025; 0.0058; 0.0027	−25; 0	−0.0043; 0.0359; −0.0305	0.0116; 0.0019; 0.0053
0; −20	0.0160; −0.0468; 0.0181	0.0054; 0.0055; 0.0039	−20; 0	0.0230; 0.0281; −0.0236	0.0144; 0.0028; 0.0040
0; −15	0.0095; −0.0401; 0.0153	0.0024; 0.0072; 0.0031	−15; 0	0.0250; 0.0192; −0.0191	0.0086; 0.0021; 0.0055
0; −10	0.0024; −0.0288; 0.0036	0.0019; 0.0041; 0.0012	−10; 0	0.0290; 0.0115; −0.0170	0.0105; 0.0014; 0.0034
0; −5	−0.0006; −0.0151; −0.0033	0.0028; 0.0066; 0.0020	−5; 0	0.0143; 0.0068; −0.0152	0.0107; 0.0024; 0.0052
0; 0	−0.0010; 0.0012; −0.0110	0.0000; 0.0000; 0.0000	0; 0	−0.0010; 0.0012; −0.0110	0.0000; 0.0000; 0.0000
0; 5	0.0008; 0.0086; −0.0145	0.0025; 0.0064; 0.0012	5; 0	−0.0159; 0.0082; −0.0037	0.0037; 0.0032; 0.0101
0; 10	0.0046; 0.0147; −0.0150	0.0025; 0.0057; 0.0008	10; 0	−0.0329; 0.0114; 0.0024	0.0072; 0.0031; 0.0100
0; 15	0.0105; 0.0173; −0.0116	0.0032; 0.0037; 0.0009	15; 0	−0.0364; 0.0169; 0.0114	0.0120; 0.0014; 0.0067
0; 20	0.0178; 0.0102; −0.0060	0.0048; 0.0041; 0.0017	20; 0	−0.0533; 0.0230; 0.0132	0.0102; 0.0015; 0.0062
0; 25	0.0274; −0.0164; 0.0088	0.0077; 0.0016; 0.0026	25; 0	−0.0567; 0.0301; 0.0153	0.0095; 0.0041; 0.0069
0; 30	0.0403; −0.0459; 0.0244	0.0073; 0.0065; 0.0029	30; 0	−0.0379; 0.0432; 0.0164	0.0112; 0.0025; 0.0064
−60; 0	-----; -----; 0.0209	-----; -----; 0.0106	35; 0	-----; -----; −0.0172	-----; -----; 0.0071
−55; 0	-----; -----; 0.0002	-----; -----; 0.0066	40; 0	-----; -----; −0.0339	-----; -----; 0.0076
−50; 0	-----; -----; −0.0111	-----; -----; 0.0076	45; 0	-----; -----; −0.0532	-----; -----; 0.0099
−45; 0	-----; -----; −0.0221	-----; -----; 0.0023	50; 0	-----; -----; −0.0592	-----; -----; 0.0072
−40; 0	-----; -----; −0.0315	-----; -----; 0.0025	55; 0	-----; -----; −0.0574	-----; -----; 0.0089
−35; 0	-----; -----; −0.0375	-----; -----; 0.0037	60; 0	-----; -----; −0.0491	-----; -----; 0.0180

**Table 5:** Pitch and roll angle for each of the positions in Figure 11.

Orientation	Pitch, θ (deg)	Roll, *ϕ* (deg)	Orientation	Pitch, θ (deg)	Roll, *ϕ* (deg)	Orientation	Pitch, θ (deg)	Roll, *ϕ* (deg)
1	0	−30	10	0	15	19	−5	0
2	0	−25	11	0	20	20	0	0
3	0	−20	12	0	25	21	5	0
4	0	−15	13	0	30	22	10	0
5	0	−10	14	−30	0	23	15	0
6	0	−5	15	−25	0	24	20	0
7	0	0	16	−20	0	25	25	0
8	0	5	17	−15	0	26	30	0
9	0	10	18	−10	0	-	-	-

## Data Availability

The original contributions presented in this study are included in the article/Supplementary Materials. Further inquiries can be directed to the corresponding author.

## Supplementary Materials

The following supporting information can be downloaded at: https://www.mdpi.com/article/10.3390/s25030835/s1, Table S1: F2-Temperature Dependence; Table S2: F3-Peaks_20240826180838; Table S3: F4a-Peaks_20240813122330; Table S4: F4b-Peaks_20240813123508; Table S5: F5to7-Caract_est_for_art_X; Table S6: F5to7-Caract_est_for_art_Y; Table S7: F8-Caract_est_for_art_X_Volt_avg; Table S8: F8-Caract_est_for_art_Y_Volt_avg; Table S9: F9-Positions_Voltaje15nov2024; Table S10: F10-Positios_Voltaje15nov2024_Sev_meas; Table S11: F12-Noise_data.
